# Single-cell multi-omics as a window into the non-coding transcriptome

**DOI:** 10.1186/s41065-025-00573-7

**Published:** 2025-09-16

**Authors:** Hua-Sheng Chiu

**Affiliations:** Department of Pediatrics, Texas Children’s Cancer Center, Baylor College of Medicine, Houston, TX 77030 USA

**Keywords:** Non-coding RNA, ceRNA, miRNA, lncRNA, Systems biology, Hermes, Cupid, LongHorn, BigHorn, Single-cell sequencing

## Abstract

This Editorial introduces my research background as the new Non-coding RNA Section Editor at Hereditas and serves as a call for submissions for the special collection, “Single-Cell Multi-Omic Analysis of Cancer Interactome”. Single-cell multi-omic approaches are opening new windows into the non-coding transcriptome, revealing how these molecules shape cellular identity in ways bulk analyses often miss. In this article, I focus on non-coding RNAs and emphasize the importance of integrative systems biology and multi-omics in this field, while also highlighting how these perspectives can guide future discoveries. I cordially invite you to contribute your work by submitting manuscripts to this collection.

## Background

### A new frontier: the non-coding transcriptome

The discovery of non-coding RNAs (ncRNAs) has added a pivotal layer of complexity to our understanding of cellular regulation [1–4]. Genome-wide annotation efforts [5] revealed that these molecules are transcribed from a substantial portion of the genome’s “dark matter”, a vast non-protein-coding region now known to be transcriptionally active. Despite outnumbering their protein-coding counterparts [6, 7]the regulatory functions and molecular targets of these non-coding molecules remain largely uncharacterized [8]. This knowledge gap is driven by three major challenges [9]. First, ncRNAs are significantly harder to quantify and identify due to their high expression specificity across the heterogeneous landscape of tissues, tumors, and individual cells, as well as their limited sequence conservation across species. Second, the intricate interplay between ncRNAs and canonical regulators, such as transcription factors and RNA-binding proteins, remains largely unknown, hindering their full integration into the larger cellular network. Third, the lack of verified interactions, relevant datasets, and predictive tools leaves researchers to solve the ncRNA puzzle from incomplete data.

**Fig. 1:**
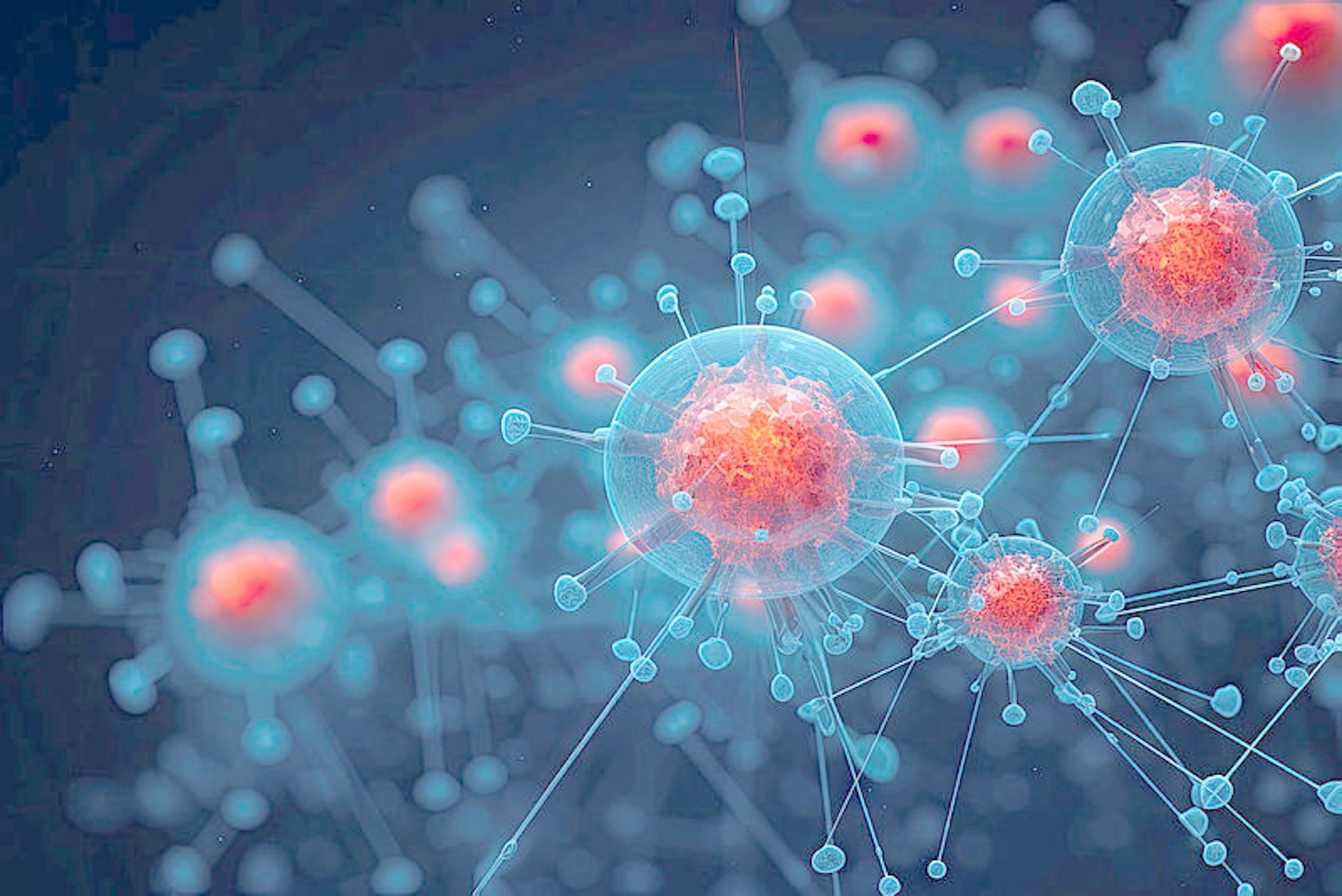
Image credit: © ratatosk / stock.adobe.com / Generated with AI

### The rise of integrative systems biology

Recent technical advances in high-throughput sequencing and multiomics data allow us to simultaneously measure genetic, transcriptomic, epigenomic, and proteomic changes at both a massive scale and single-cell resolution [10, 11]. However, this data tsunami’s sheer volume and analytical complexity make it impossible to proceed without the aid of modern computational tools like data mining, machine learning, and artificial intelligence [12]. This presents a unique opportunity for systems biology to develop powerful integrative algorithms that can combine and interpret these massive datasets across multiple regulatory modalities [13, 14]. By integratively reverse-engineering molecular interactomes from multiomics data, these efforts enable a shift from single-component studies to a network-based perspective, yielding previously unattainable biological insights. Building on this success, integrative systems biology is uniquely positioned to unlock a significant portion of unexamined multiomics data relevant to ncRNA biology.

## Our contribution to non-coding RNA research

### A systems biologist’s journey into the ncRNA universe

Over the past years, our work has pushed the boundaries of ncRNA research by elucidating their regulatory potential. This endeavor fills a critical knowledge gap, as these molecules are often overlooked in comprehensive datasets, limiting our understanding of their roles in cellular networks. Our approach is based on the principle that a regulator’s cellular function is dictated by its targets and interacting partners [15–17]. Accordingly, developing advanced algorithms to accurately infer target sets will improve our confidence in defining a regulator’s function. Toward this goal, we leveraged publicly available patient-, cancer cell-, and tissue-specific multiomics profiles from large-scale cohorts, including TCGA [18]TCPA [19]CCLE [20]LINCS [21, 22]DeepMap [23]ENCODE [24]and RNA Atlas [16]. These profiles were used to systematically identify ncRNAs that are differentially altered or respond to perturbagens such as siRNAs [25]shRNAs [26]CRISPRa [27]CRISPRi [28–30]ASOs [7]and FDA-approved small molecules [20, 21]. This analysis then searches for targets whose abundance is highly correlated with these dynamic changes, and investigates whether these targets are enriched in key biological pathways as a surrogate to infer ncRNA functions [31].

Our past research specifically focused on two major classes of ncRNAs, namely microRNAs (miRNAs), which are approximately 22 nucleotides long, and long non-coding RNAs (lncRNAs), which are over 200 nucleotides long. Leveraging integrative systems biology approaches, we inferred their molecular targets and functions [16] in diseases like cancers [32–37]. These efforts integrated ncRNA interactions into existing networks and related them to canonical regulators, which not only provided a more comprehensive understanding of cellular regulation but also successfully assigned functions to the majority of these previously uncharacterized ncRNAs, bringing them into the scientific spotlight.

### Hermes and Cupid: charting a new regulatory horizon for miRNAs

We developed the Hermes algorithm [38] to map the competing endogenous RNA (ceRNA) [31, 39] network using mutual information [40]. This approach revealed a novel regulatory mechanism in which RNA transcripts compete for the binding of common miRNAs through a titration or ‘sponge’ effect. Our analysis strikingly showed that this network is as extensive as other established regulatory mechanisms, such as transcription factor-target networks. We also demonstrated that this type of regulation not only facilitates crosstalk between key driver genes and pathways in cancers, but it also provides a potential explanation for tumors that develop without obvious genomic variability [41, 42]including copy number alternations and differential methylation.

Inspired by these observations, we developed the Cupid algorithm [43] to enhance the accuracy of miRNA target predictions. This algorithm operates on the core principle that competition among targets for a shared miRNA provides stronger evidence that these are genuine miRNA-target interactions (true positives). Taking advantage of Cupid’s improved predictive power [41]we also uncovered a unique phenomenon that is when two targets are regulated by a large pool of common miRNA regulators, they are more likely to engage in ceRNA-mediated cross-regulation across multiple tumor types or cellular contexts [42]. In addition, it has been previously established that the let-7 miRNA family comprises two distinct subfamilies, characterized by the presence or absence of a cold shock domain-binding site. Our analysis of Cupid’s predicted targets confirmed the differential regulation of these two subfamilies by the LIN28 protein in cancers, as evidenced by the distinct coexpression patterns observed between LIN28 and their Cupid-inferred targets [44]. Both the Hermes and let-7 studies were featured as journal cover stories upon publication.

### LongHorn and BigHorn: decoding lncRNA functions through target analysis

Our work with the TCGA Pan-Cancer Atlas project [18] led to the development of LongHorn [15]an algorithm designed to expand our target prediction landscape to lncRNAs. By integrating four established lncRNA regulatory mechanisms (guide, co-factor, decoy, and switch), LongHorn inferred lncRNA targets from over ten thousand patient profiles and classified their regulatory modalities as either transcriptional or post-transcriptional. As one of the few integrative systems biology approaches for genome-wide lncRNA target predictions, we applied LongHorn to the RNA Atlas [16]a large-scale sequencing project that profiles RNA expressions in over 300 normal tissues using bulk RNA-sequencing with and without PolyA selection and small RNA sequencing. This enabled us to infer targets and assign key functions to novel ncRNAs, including thousands of single-exon lncRNAs and hundreds of circRNAs not previously characterized. All findings and predictions are publicly available through the R2 web portal [16].

Our most recent effort is the development of the BigHorn algorithm [17] to enhance predictions of lncRNA transcriptional targets. For this work, we investigated flexible binding motifs within the proximal promoters of target genes and correlated the presence of these motifs with TCGA pan-cancer expression profiles to identify highly predictive patterns and their corresponding targets. The analysis revealed a significant enrichment of BigHorn-inferred transcriptional targets within genes and their promoter binding sites within DNA regions affected by lncRNA perturbation. Strikingly, we identified hundreds of targets coordinately regulated by the same lncRNAs across both transcriptional and post-transcriptional modalities. These targets exhibit a tight coupling, or highly correlated expression patterns, with their regulated lncRNAs. This unexpected observation offers a novel insight into how lncRNAs can coordinate the regulation of their targets through multiple regulatory layers and integrate themselves into larger cellular networks, working in conjunction with canonical regulators to orchestrate cellular dynamics. This work was recognized on the journal cover upon publication.

### Driving ncRNA discoveries for global impact

While developing our systems biology algorithms, we identified a clear path to translate our ncRNA research into clinical applications [45, 46]. This strategic shift has motivated us to collaborate closely with medical and radiation oncologists to ensure that our findings have a direct impact on patient care. These partnerships have already yielded significant discoveries with high therapeutic potential. For example, we have identified several lncRNAs that can serve as biomarkers for early-stage cancer diagnosis and to predict therapeutic response to cancer treatment [15]. We have also uncovered a subset of lncRNAs that can modulate the DNA damage response (DDR) pathway in cancer cells. We have observed that targeting these molecules significantly compromises a cancer cell’s ability to recover from radiation [17]. This discovery has led to our most promising application, which is a developing synthetic lethality approach for pediatric sarcoma patients. By silencing a specific lncRNA in patients with mutations in a DDR protein, we aim to increase the tumor’s sensitivity to radiotherapy and improve the patient response to therapy with lower doses and less toxicity. Ultimately, the translational models we have developed for cancer, once proven successful, can be applied to other diseases to improve global well-being. This approach directly contributes to the United Nations’ Sustainable Development Goal 3, Good Health and Well-Being, and aligns with its long-term ambition to reduce premature deaths through prevention and treatment by 2030 [47].

## About the collection

### When the generalist meets the specialist

Bulk sequencing has long dominated the systems biology field, leading to the development of powerful algorithms and tools for the rapid and effective integration and interpretation of multiomics data. With the emergence of single-cell sequencing, however, the focus of cancer research has shifted dramatically, with researchers now pouring their resources into this new method of data generation. These new profiling techniques are completely revitalizing cancer research by enabling the investigation of complex topics, such as cellular heterogeneity, novel or rare cell populations, and the dynamics of cell-cell communication and evolution [10]. They also provide a deeper understanding of how distinct cell types contribute to the therapeutic resistance through perturbation studies [48].

Despite its widespread applications, single-cell sequencing still faces fundamental issues, including high cost, intense technical complexity, limited data quality, a high dropout rate, and a lower number of genes detected per cell [12, 49, 50]. For this reason, single-cell analysis will not immediately replace its bulk counterparts in clinical applications. Indeed, the two profiling technologies will remain complementary and synergetic in the near term: bulk sequencing (the generalist) provides a broad, population-averaged measure of the entire gene spectrum, while single-cell sequencing (the specialist) offers a focused, cell-specific analysis of marker genes with intricate detail. A successful example of their collaboration is the active research field focused on leveraging single-cell sequencing to deconvolute bulk sequencing profiles, or vice versa [51].

### Integrative systems biology in the single-cell era

The latest trend in single-cell profiling has, not surprisingly, created a second data tsunami, which presents an urgent need for systems biologists to develop a next-generation suite of algorithms and tools [52]. From our perspective, proposing a collection with a focus on this pressing need can be an effective way to highlight the novel solutions to this data challenge. In this collection, we therefore urge submissions that focus on the integrative study of single-cell/nucleus RNA-seq (scnRNA-seq) and single-cell ATAC-seq (scATAC-seq) for two reasons: these are the most abundant types of single-cell profiles, and there is a high demand for approaches that can combine evidence from both data types for deeper interpretation and insights. We also welcome studies that incorporate joint analysis of bulk sequencing profiles at any stage of your work and will prioritize those that leverage landmark or atlas-level datasets. The scope of these integrative studies can encompass protein-coding genes, ncRNAs, or a combination of the two. For more information, please visit the collection webpage.

## Conclusions

Emerging evidence suggests that ncRNAs play a key role in virtually all aspects of cellular regulation. They can drive complex biological phenomena by coordinating regulation across modalities, either alone or in cooperation with canonical regulators. The recent rise in popularity of single-cell multi-omics is expanding the research arsenal, enabling the investigation of complex biological systems and disease mechanisms. We anticipate that developing novel computational solutions to integrate these profiles will revolutionize ncRNA research. These tools will help reveal new roles for ncRNAs in specific cellular processes beyond the reach of bulk sequencing, such as cell-cell communication and cell evolution. In the long term, we foresee that these findings will deepen our understanding of human biology, provide a solid foundation for new therapeutic applications or strategies, and ultimately improve public health and quality of life. Accordingly, this special collection is a catalyst for new integrative studies in the single-cell era, serving as a vital step toward achieving these long-term goals.

## Data Availability

Data sharing is not applicable as this work did not generate or analyze any datasets.

## Abbreviations

ncRNA: Non-coding RNA
microRNA: miRNA
lncRNA: Long non-coding RNA
ceRNA: Competing endogenous RNA
DDR: DNA damage response
scnRNA-seq: Single-cell/nucleus RNA-seq
scATAC-seq: Single-cell ATAC-seq
